# Aortocaval Fistula: A Rare Complication of an Infrarenal Aortic Aneurysm

**DOI:** 10.5334/jbsr.3099

**Published:** 2023-04-24

**Authors:** Felix Delbare, Benjamin Leenknegt, Piet Vanhoenacker

**Affiliations:** 1 UZ Ghent, BE; 2 AZ Sint-Lucas Ghent, BE

**Keywords:** aortocaval fistula, aortic abdominal aneurysm, aortic rupture, peri-aortic hematoma, CT angiography

## Abstract

**Teaching Point:** Aortocaval fistula is a rare complication of infrarenal aortic aneurysms.

## Case History

A 74-year-old man presented to the emergency department after an episode of dyspnoea. Over the last two days, the patient had experienced continuous sharp lumbar back pain. He also had nausea with vomiting, diarrhoea, and had suffered four episodes of syncope. The patient had a history of arterial hypertension, treated with ACE inhibitors. Clinical examination revealed abdominal pain on palpation and a pulsatile abdominal mass. Tachycardia and arterial hypotension were noted. Peripheral blood analysis showed elevation of lactate.

**Figure 1:**
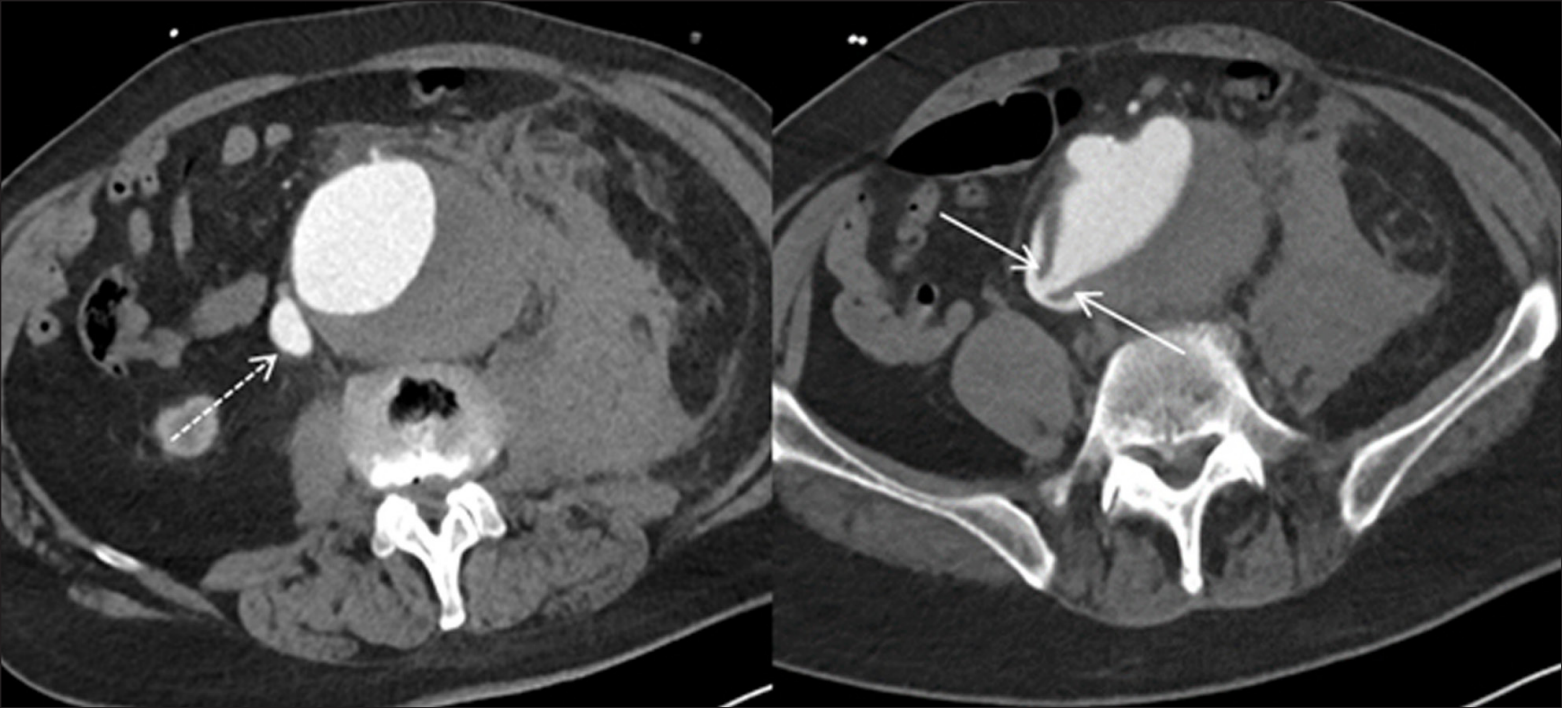


A multi-phase thoraco-abdominal computed tomography (CT) scan was requested. On CT, there was a large infrarenal aortic aneurysm up to 10 cm with a large para-aortic hematoma. Arterial phase opacification of the inferior vena cava and the common iliac vein, especially on the right side, was present (Figure 1, dashed arrow). Detailed study of the scan revealed a linear connection between the aorta aneurysm and the inferior vena cava (Figure 1, arrows), suggestive of an aortocaval fistula.

**Figure 2:**
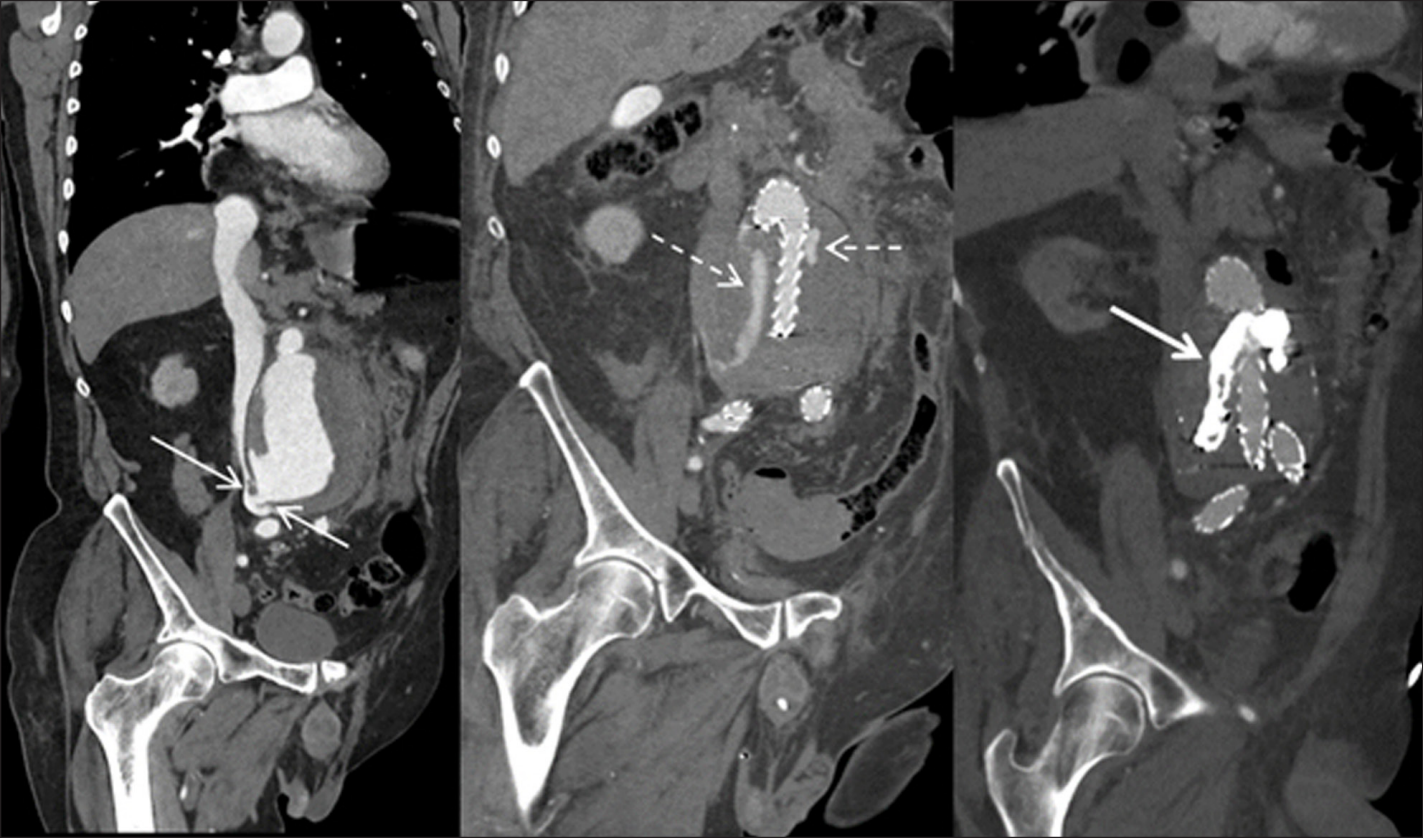


**Figure 3:**
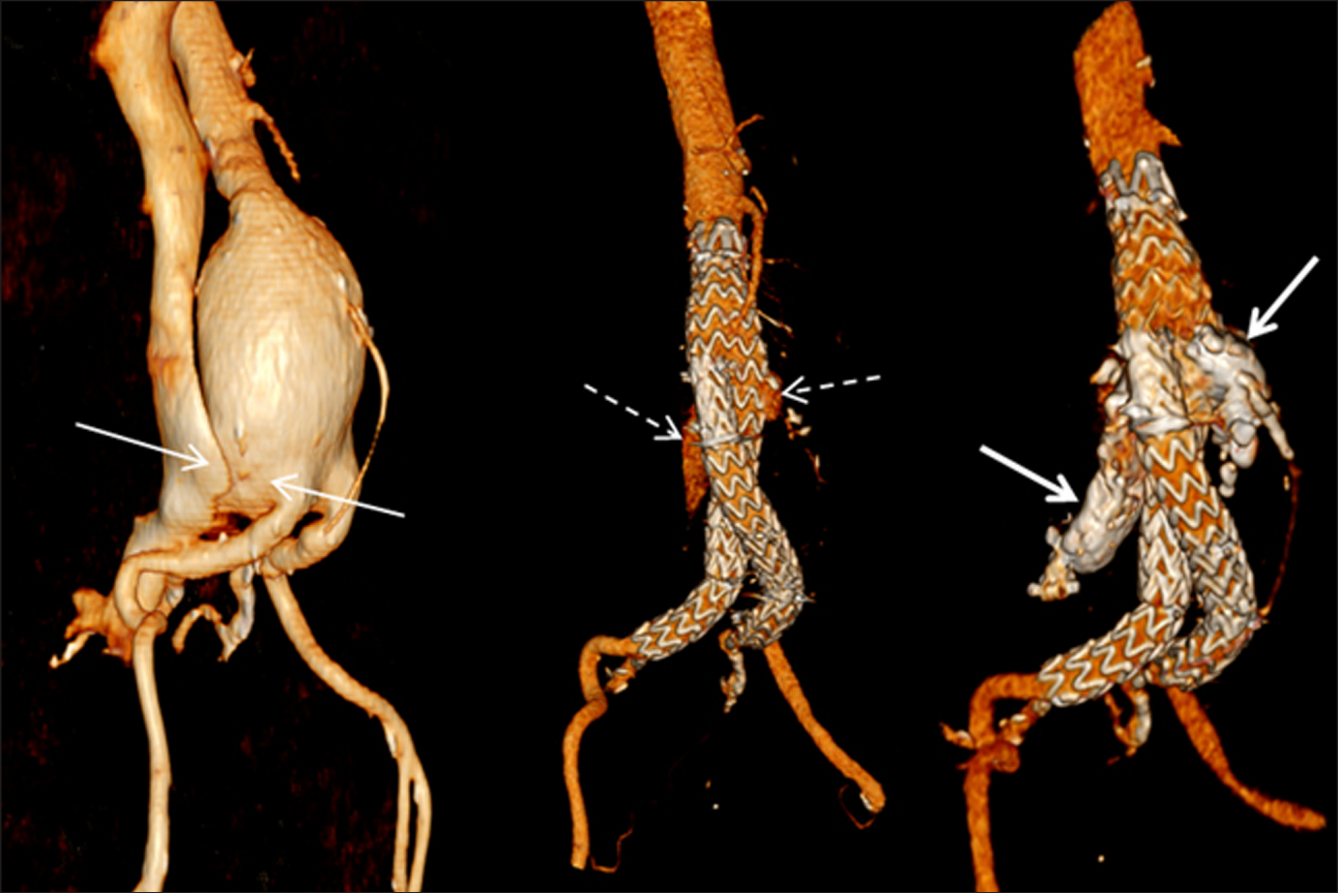


Prompt endovascular aortic repair was performed. On a post-surgery CT scan, an endoleak type 1 was depicted. The leak was subsequentially embolized with cyanoacrylate glue (Figures 2 and 3: arrows ACF, dashed arrows endoleak, bold arrows cyanoacrylate glue).

## Comments

Aortocaval fistula (ACF) is a rare complication of a chronically eroding abdominal aortic aneurysm in which an arteriovenous connection between the aorta and the inferior vena cava develops. The most frequent causes of ACF are (ruptured) aortic abdominal aneurysm (80%), traumatic injury (15%), and iatrogenic lesion (5%; e.g., exploratory laparotomy and lumbar laminectomy). A minority of cases is related to mycotic aneurysms, connective tissue diseases and Takayasu’s arteritis. Unfortunately, due to its indistinct signs and symptoms and rare occurrence, the diagnosis is often delayed with potential lethal outcome [1].
